# An Interesting Case of Hypogammaglobulinemia Caused by the Combined Effect of Rituximab Infusion and Common Variable Immunodeficiency (CVID)

**DOI:** 10.7759/cureus.72108

**Published:** 2024-10-22

**Authors:** Manish Gaba, Dipshi Jain, Naveen Kumar, Ankita Pandey, Arun Dewan

**Affiliations:** 1 Internal Medicine, Max Smart Super Speciality Hospital, New Delhi, IND

**Keywords:** common variable immunodeficiency deficiency, fungal pneumonia, hypogammaglobulinemia, hypoglobulinemia, rituximab therapy

## Abstract

This case report is about a patient who presented with bilateral fungal pneumonia. He had a background of viral hepatitis-E infection five years ago, non-Hodgkin lymphoma in remission for four years, and a severe COVID-19 infection three years ago. He was found to have a common variable immune deficiency (CVID) in the background of long-standing hypoglobulinemia, which had never been evaluated. The patient had a history of rituximab infusion for the management of non-Hodgkin lymphoma, which likely worsened his hypogammaglobulinemia. This is an interesting case report that discusses hypogammaglobulinemia in the background of CVID and rituximab infusion.

## Introduction

Hypogammaglobulinemia, as the name implies, refers to low serum immunoglobulin levels. It is the most common primary immunodeficiency [1]. The age distribution is from six to 10 years and 20 to 40 years, showing a bimodal incidence [1]. This condition predisposes them to recurrent infections, allergies, neoplasms, and autoimmunity. Common variable immunodeficiency (CVID) is the most common etiology of hypogammaglobulinemia in adults [2]. The primary causes of immunodeficiencies consist of genetic disorders and chromosomal anomalies. Secondary causes include steroids, immunosuppressant drugs, nutritional disorders, infections, chemotherapy, malignancy, nephrotic syndrome, and metabolic diseases [3]. This case report highlights the importance of both primary and secondary causes of hypogammaglobulinemia.

## Case presentation

A male in his 40s came to the emergency department with a background of acute hepatitis-E infection five years ago, non-Hodgkin’s lymphoma in remission four years ago, and a severe COVID-19 infection three years back. He had no history of foreign travel. He belonged to a middle-income socioeconomic group working in a corporate job. He had complaints of fever with chills, associated with an evening rise of temperature to 101°F, subsiding with a tablet of paracetamol. He had complaints of persistent cough with white-colored sputum which was moderate amount in quantity and was non-blood tinged. He had a history of admission at another center two months ago with similar complaints. The computed tomography (CT) scan of the chest done at that time revealed multiple, discrete, and confluent centrilobular nodules in bilateral lung parenchyma with tree-in-bud appearance with patchy areas of consolidation with ground glass opacities in the superior segment of the bilateral upper lobe. The CT scan of the abdomen showed a few enlarged pre-aortic and para-aortic lymph nodes with mild post-contrast enhancement. He was taken up for a bronchoscopy, which had findings suggestive of pneumonia. Bronchoalveolar lavage (BAL) showed elevated galactomannan, thus suggesting a fungal infection, for which he was treated with voriconazole. However, after completing the course of treatment for 12 weeks, his fever and cough recurred. He subsequently came to our center for further management. His physical examination revealed a heart rate of 90 beats per minute, blood pressure of 120/80 mmHg, and oxygen saturation of 96% on room air. He was febrile, more during evening hours, with temperatures going up to 101-103°F. There were no palpable lymph nodes. Respiratory system examination revealed reduced air entry in the right base, with crepitations present in bilateral bases. There was throat congestion and maxillary sinus tenderness. Otorhinolaryngology examination revealed throat congestion with granulations in the posterior pharyngeal wall. His abdomen examination revealed hepatosplenomegaly. His cardiovascular and neurological examination was normal.

**Figure 1:**
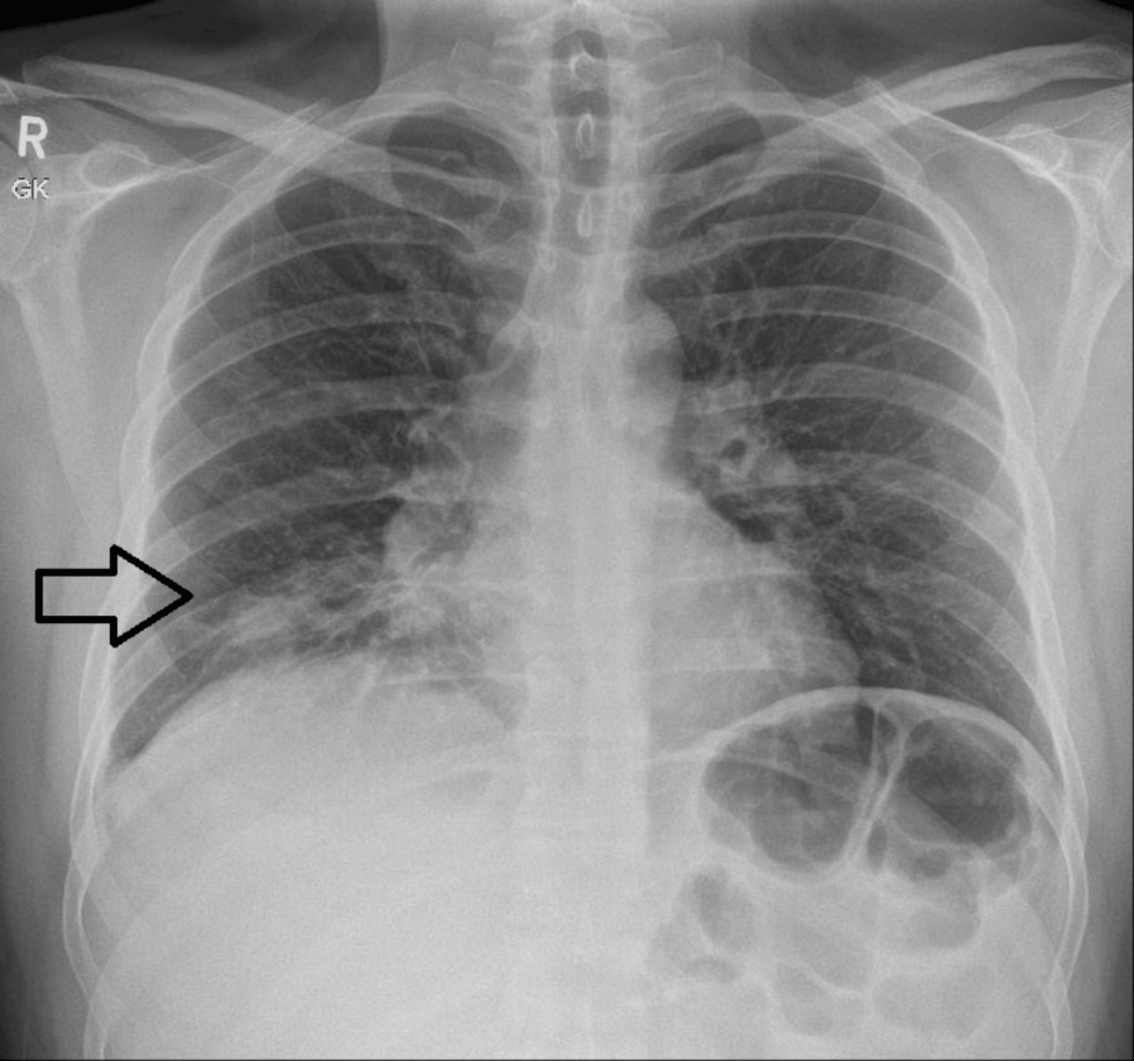
Chest radiograph in posteroanterior view shows inhomogeneous patchy opacity in the right lower zone.

His blood investigation revealed mild leukocytosis (Table 1). Biochemistry revealed hypoalbuminemia and hypoglobulinemia. Procalcitonin was significantly elevated. His tests for viral pathologies were negative for Epstein-Barr virus (EBV), COVID-19, swine flu (H1N1), influenza A, influenza B, and respiratory syncytial virus (RSV). He tested negative for hepatitis-C virus IgG (HCV IgG), hepatitis B antigen (HBsAg), and HIV-I and II. The thyroid profile was within normal limits. The chest radiograph revealed inhomogeneous patchy opacity seen in the right lower zone (Figure 1).

**Table 1:** Laboratory investigation EBV: Epstein-Barr virus; PCR: polymerase chain reaction; IgG: immunoglobulin G, IgM: immunoglobulin M, IgA: immunoglobulin A; RSV: respiratory syncytial virus; HCV: hepatitis C virus, HIV: human immunodeficiency virus, HbsAg: hepatitis B surface antigen, M. TB: *Mycobacterium tuberculosis*, BAL: bronchoalveolar lavage, Procal: procalcitonin; TSH: thyroid stimulating hormone

Investigations	Values	Reference ranges
Hemoglobin (g/Ll)	12.5	13–17
Total leucocyte count (cell/cum)	10.3	4–10
Platelet (cell/cumm)	181	150-410
Creatinine (mg/dL)	0.8	0.9–1.3
Sodium (mmol/L)	139	136–146
Potassium (mmol/L)	4.4	3.5–5.1
Calcium (mg/dL)	9.5	8.8–10.2
Aspartate aminotransferase (IU/L)	43	15-41
Alanine aminotransferase (IU/L)	20	17-63
Alkaline phosphatase (IU/L)	66	32-91
Gamma-glutamyl transferase (IU/L)	62	7-50
Globulin	1.8	2.3-3.5
Albumin (g/dL)	3.1	3.5-5.0
EBV, IgG	<10.0	Neg - <20.0
EBV, IgM	<10.0	Neg - <20.0
EBV DNA PCR (copies /ml)	<157	<157 – negative
APTT (sec)	34.8	23.7-33.5
INR ( %)	1.17	sec
IgG ( mg/dl )	<75.0	791- 1643
IgM( mg/dl )	<20.0	43-279
IgA ( mg/dl )	<10.0	83- 406
COVID-19 PCR	Negative	Negative
Influenza A	Negative	Negative
Influenza B	Negative	Negative
H1N1	Negative	Negative
RSV	Negative	Negative
Influenza A (H3N2)	Negative	Negative
HCV IgG (s/co)	0.01	<0.90
HIV (I and II)	Negative	Negative
HBsAg (s/co)	0.10	<0.90
Galactomannan	2.21	<0.5
M.TB PCR	Negative	Negative
Galactomannan BAL	1.0	<0.5
Lower respiratory panel BAL	Negative	Negative
AFB culture BAL	Negative	Negative
BAL culture and sensitivity	Negative	Negative
BAL fungal culture	Negative	Negative
BAL gram stain	Occasional gram-positive cocci in pairs	Negative
Procal (ng/ml)	84.87	<0.50
TSH (uIL/ml)	1.86	0.34-5.6

**Figure 2:**
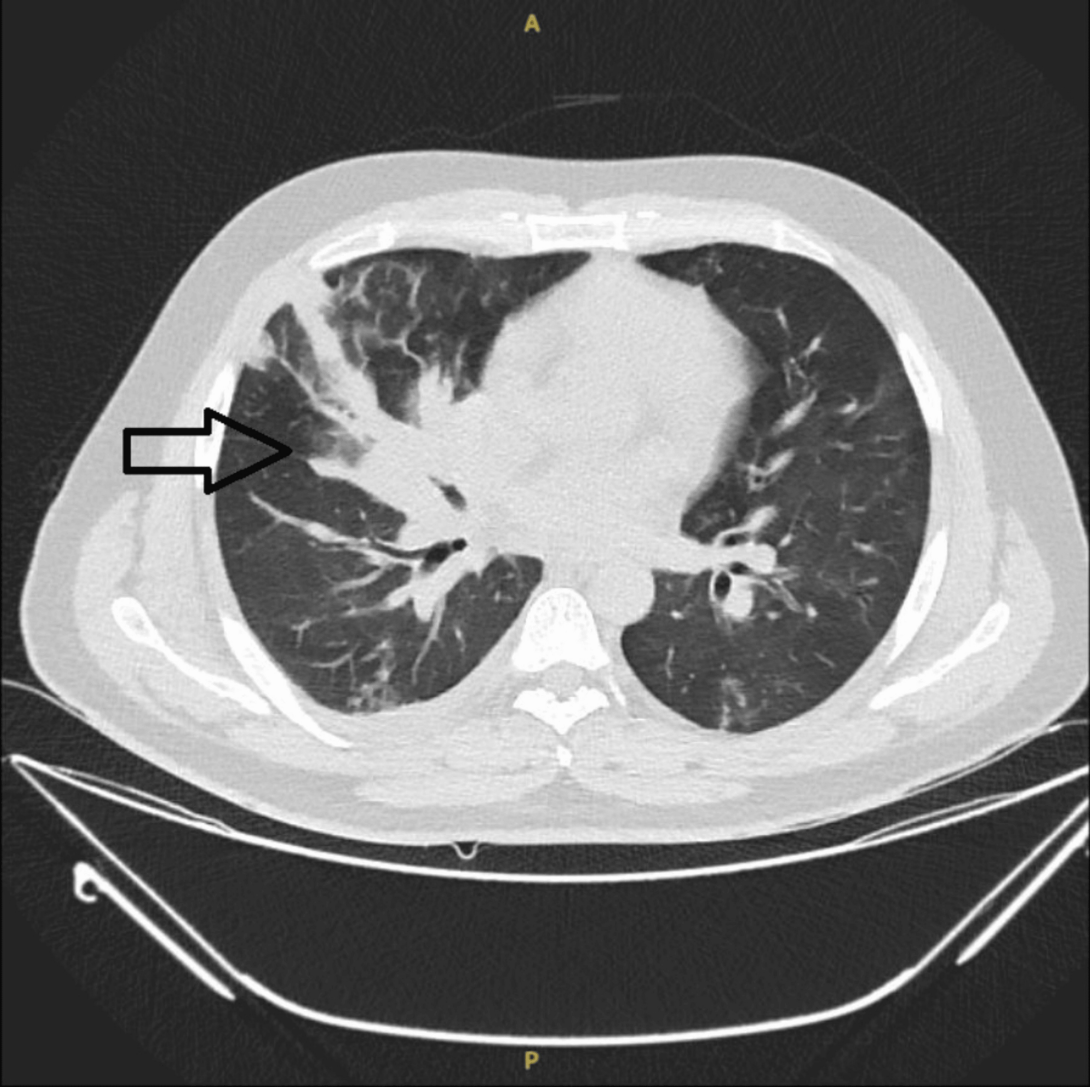
Computed tomography of the chest shows confluent airspace opacities in the right middle lobe and basal segments of the right lower lobe, suggestive of pneumonia with mild synpneumonic right pleural effusion. Also, there are multiple patchy areas of ground glass opacities scattered in both lungs with mild bronchial wall thickening.

An ultrasound of the abdomen revealed hepatosplenomegaly. High-resolution computed tomography (HRCT) of the thorax showed confluent airspace opacities in the right middle lobe and basal segments of the right lower lobe, suggestive of pneumonia with mild syn-pneumonic right pleural effusion. Also, there were multiple patchy areas of ground glass opacities scattered in both lungs with mild bronchial wall thickening (Figure 2). 

**Figure 3:**
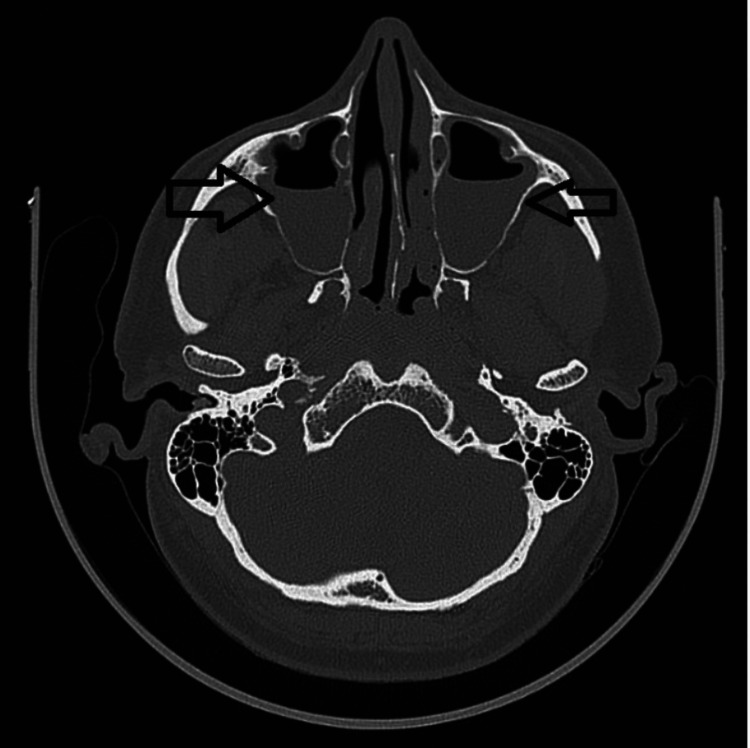
Computed tomography of the paranasal sinus shows pansinusitis with air fluid levels in bilateral maxillary sinuses and obliterated bilateral osteomeatal complexes and partially obliterating left frontonasal recess.

An otorhinolaryngology opinion was taken, and his nasal endoscopy was done, which showed mucoid to mucopurulent discharge in the bilateral middle meatus with bilateral inferior turbinate hypertrophy. A non-contrast computer tomography (NCCT) of the paranasal sinus (PNS) was advised, which revealed pansinusitis with air-fluid levels in bilateral maxillary sinuses and obliterated bilateral osteomeatal complexes and partially obliterated left frontonasal recess (Figure 3).

The patient was taken up for bronchoscopy, and the BAL samples were sent for analysis. The Gram stain revealed occasional Gram-positive cocci in pairs. His serum and BAL galactomannan were found to be high. All other BAL reports were within normal limits (Table 1). The hypersensitivity pneumonitis panel showed no abnormality. So, the patient had pneumonia with pansinusitis, likely fungal in etiology in view of raised galactomannan. When we reviewed his history, he had a history of viral hepatitis E infection, which took months to resolve. Subsequently, he had a history of non-Hodgkin lymphoma for which he took treatment and was currently in remission. He had a history of a severe COVID-19 infection. His past records were re-evaluated. His annual health insurance blood work showed hypoglobulinemia. At the time of his admission for hepatitis E infection, hypoglobulinemia was present. This raised the possibility of CVID. He had been treated with rituximab for management of non-Hodgkin lymphoma four years ago. Rituximab is a known cause of hypogammaglobulinemia. We sent his immunoglobulin A, G, and M levels, and all were found to be very low (Table 1). This was suggestive of hypogammaglobulinemia. It was due to CVID, which got worse with the use of rituximab.

Differential diagnosis

This was a young male in his 40s presenting with fungal pneumonia and pansinusitis, which was not improving despite being on antifungal therapy. He was not diabetic, his HIV test was negative, and there were no other known comorbidities. A review of his records showed persistent hypoglobulinemia. This raised the possibility of CVID. He had been treated with rituximab for management of non-Hodgkin lymphoma. Rituximab is known to cause hypogammaglobulinemia. Persistent hypoglobulinemia was a clue to the possibility of CVID in the past, which was never evaluated. The administration of rituximab likely worsened this pre-existing condition.

Treatment

The patient was started on empirical intravenous antibiotics, which consisted of piperacillin+ tazobactam as per local sensitivity guidelines and other supportive measures. He developed a high-grade fever of up to 103°F and was started on clarithromycin for atypical cover. His serum galactomannan was elevated, and he started on voriconazole. His condition was not improving, and he was started on meropenem empirically. The BAL gram stain showed occasional Gram-positive cocci, and we initiated him on teicoplanin. Despite all these measures, he continued to be sick. He was subsequently diagnosed as having hypogammaglobulinemia. He was started on intravenous immunoglobulin (IVIG) therapy. His symptoms improved remarkably within 48 hours. He was given a second dose of IVIG after six days of the primary dose, following which his IgG level improved to 517 mg/d. He was subsequently discharged in stable condition and was advised to follow up with repeat IgG levels.

Follow-up

The patient is on regular follow-up with the internal medicine department. Symptoms have improved with no episodes of recurrent fever. He was administered IVIG six weeks later based on his IgG levels. The plan is to keep on maintenance IVIG therapy guided by his IgG levels.

## Discussion

Common variable immunodeficiency is a disorder of defective B cell function that leads to impaired immunoglobulin production [4]. The clinical manifestations include recurrent infections, chronic lung disease, autoimmune disorders, gastrointestinal disease, or increased risk of malignancy [4]. It is the most frequent symptomatic primary immunodeficiency disorder worldwide [4]. Common variable immune deficiency affects approximately one in 25,000 individuals [4]. It is more prevalent in northern Europe [4]. It is usually diagnosed between the ages of 20 and 45 [4]. Environmental and genetic factors are implicated in the pathogenesis. The environmental triggers are not known. The genetic influence (Table 2) in CVID is due to an intrinsic B cell defect, an intrinsic T cell defect, and mutations in tumor necrosis factor (TNF) receptors [4]. However, CVID can be present without a known genetic defect. 

**Table 2:** Genetic defects commonly seen in CVID TNF: tumor necrosis factor; CD19: cluster of differentiation 19; ICOS: inducible co-stimulator; TACI: transmembrane activator and CAML interactor; BAFFR: B-cell activating factor receptor; MSH5: mutS homolog 5 This table has been created by the author and the data for this table has been adapted from the book "Common Variable Immunodeficiency" Copyright © 2024, StatPearls Publishing LLC [4]. This book is distributed under the terms of the Creative Commons Attribution-NonCommercial-NoDerivatives 4.0 International (CC BY-NC-ND 4.0 (http://creativecommons.org/licenses/by-nc-nd/4.0/)

Defects	Genes involved
Intrinsic B cell defects	CD19-deficiency by mutations in CD19; 16p11.2
Intrinsic T cell defects	ICOS-deficiency by mutations in ICOS; 2q33
Mutation in TNF receptors	TACI-deficiency or BAFFR- deficiency by mutations in TNFRSF13B and TNFRSF13C respectively; 17p11.2 and 22q13.1-q13.31
Monogenic defects	MSH5, CD81, and CD20 deficiencies

Chapel et al. have delineated five clinical phenotypes for CVID (Table 3) [5].

**Table 3:** The clinical phenotypes of common variable immune deficiency (CVID) This table has been created by the author and the data for this table has been adapted from the article "Common variable immunodeficiency disorders: division into distinct clinical phenotypes" by Chapel et al. [5]

Clinical phenotypes of common variable immune deficiency (CVID)
Uncomplicated (patients usually suffer only infections)
Autoimmunity
Polyclonal lymphocytic infiltration (this includes lymphoid interstitial pneumonitis, unexplained granulomas, unexplained hepatomegaly and/or splenomegaly, and lymphadenopathy)
Enteropathy (biopsy-proven lymphocytic infiltration in lamina propria and interepithelial mucous with villous atrophy)
Lymphoid malignancy

Rituximab is an anti-CD20 chimeric antibody with human IgG1 immunoglobulin constant regions and variable regions from an anti-CD20 murine antibody [6]. CD20 is the target antigen of rituximab. It is a surface transmembrane protein marker that is expressed in B cells. This is predominantly seen during their differentiation from the pre-B cell to plasma cells. Rituximab binds to the CD20-positive cells and induces cell death. This is mediated by various mechanisms, primarily including antibody-dependent cell-mediated cytotoxicity (ADCC), complement-mediated cytotoxicity (CDC), and antibody-dependent phagocytosis (ADP). It is a part of the treatment protocol for non-Hodgkin lymphoma [6]. Clinically significant hypogammaglobulinemia is a well-reported entity in a high proportion of patients who have been treated with rituximab [7].

Our patient had a history of hepatitis E infection, which took a long time to recover. His blood work done as a part of an annual insurance check-up showed hypoglobulinemia. The patient also had a history of non-Hodgkin lymphoma, which is seen in cases of CVID. He had been treated with rituximab for management of non-Hodgkin lymphoma. It is quite likely that the patient had a background of CVID, which had worsened after the administration of rituximab. He had not undergone a workup for baseline immunoglobulin levels before the administration of rituximab. Now he was admitted with fungal pneumonia and pansinusitis, which was not resolving despite adequate therapy. The patient was started on IVIG infusion, and he had a remarkable improvement: he became afebrile and improved symptomatically within 48 hours. The primary management of CVID is the administration of IVIG [4].

This is indicated for patients who have IgG levels that are two standard deviations below the normal range. The initial dose for IVIG is 300 to 600 mg/kg every three to four weeks [4]. The monitoring of IgG levels should take place every six months.

It is important to check baseline immunoglobulin levels prior to administration of rituximab. These levels should be monitored post rituximab infusion as well. The administration of IVIG for hypogammaglobulinemia can improve the outcome of any acquired infections in these patients.

## Conclusions

Common variable immune deficiency is an underreported entity. The evaluation and diagnosis have been streamlined in the guidelines, but they still need to be noticed. All patients should have immunoglobulin levels checked before rituximab infusion for any indication. We must look at acquired and inherited immunodeficiency as a cause for patients with infectious diseases that do not improve despite the best treatment. In this patient, it was a combined effect of rituximab infusion and CVID, which led to his prolonged disease.

## References

[1] Huq ME, Bhatnagar NK, Hostoffer RW (2024). Hypogammaglobulinemia. https://www.ncbi.nlm.nih.gov/books/NBK563134.

[2] Hsu J, Opoka R, Lund TC (2015). Hypogammaglobulinemia in sub-Saharan Africa: a case report and review of the literature. Afr Health Sci.

[3] Pimenta FM, Palma SM, Constantino-Silva RN, Grumach AS (2019). Hypogammaglobulinemia: a diagnosis that must not be overlooked. Braz J Med Biol Res.

[4] Pescador Ruschel MA, Vaqar S (2024). Common Variable Immunodeficiency. https://www.ncbi.nlm.nih.gov/books/NBK549787.

[5] Chapel H, Lucas M, Lee M (2008). Common variable immunodeficiency disorders: division into distinct clinical phenotypes. Blood.

[6] Hanif N, Anwer F (2024). Rituximab. https://www.ncbi.nlm.nih.gov/books/NBK564374/.

[7] Tieu J, Smith RM, Gopaluni S (2021). Rituximab associated hypogammaglobulinemia in autoimmune disease. Front Immunol.

